# Retinal Input Is Required for the Maintenance of Neuronal Laminae in the Ventrolateral Geniculate Nucleus

**DOI:** 10.1523/ENEURO.0022-24.2024

**Published:** 2024-09-03

**Authors:** Katelyn Stebbins, Rachana Deven Somaiya, Ubadah Sabbagh, Parsa Khaksar, Yanping Liang, Jianmin Su, Michael A. Fox

**Affiliations:** ^1^Fralin Biomedical Research Institute at Virginia Tech Carilion, Roanoke, Virginia 24016; ^2^Graduate Program in Translational Biology, Medicine, and Health, Virginia Tech, Blacksburg, Virginia 24061; ^3^Virginia Tech Carilion School of Medicine, Roanoke, Virginia 24016; ^4^Department of Molecular and Cell Biology, University of California Berkeley, Berkeley, California 94720; ^5^McGovern Institute for Brain Research, Department of Brain and Cognitive Sciences, Massachusetts Institute of Technology, Cambridge, Massachusetts 02138; ^6^School of Neuroscience, College of Science, Virginia Tech, Blacksburg, Virginia 24061; ^7^Department of Biological Sciences, College of Science, Virginia Tech, Blacksburg, Virginia 24061; ^8^Department of Biology, College of Natural Sciences, University of Massachusetts Amherst, Amherst, Massachusetts 01003

**Keywords:** GABAergic neuron, lateral geniculate nucleus, retina, retinal ganglion cells, visual thalamus

## Abstract

Retinal ganglion cell (RGC) axons provide direct input into several brain regions, including the dorsal lateral geniculate nucleus (dLGN), which is important for image-forming vision, and the ventrolateral geniculate nucleus (vLGN), which is associated with nonimage-forming vision. Through both activity- and morphogen-dependent mechanisms, retinal inputs play important roles in the development of dLGN, including the refinement of retinal projections, morphological development of thalamocortical relay cells (TRCs), timing of corticogeniculate innervation, and recruitment and distribution of inhibitory interneurons. In contrast, little is known about the role of retinal inputs in the development of vLGN. Grossly, vLGN is divided into two domains, the retinorecipient external vLGN (vLGNe) and nonretinorecipient internal vLGN (vLGNi). Studies previously found that vLGNe consists of transcriptionally distinct GABAergic subtypes distributed into at least four adjacent laminae. At present, it remains unclear whether retinal inputs influence the development of these cell-type–specific neuronal laminae in vLGNe. Here, we elucidated the developmental timeline for these laminae in the mouse vLGNe, and results indicate that these laminae are specified at or before birth. We observed that mutant mice without retinal inputs have a normal laminar distribution of GABAergic cells at birth; however, after the first week of postnatal development, these mutants exhibited a dramatic disruption in the laminar organization of inhibitory neurons and clear boundaries between vLGNe and vLGNi. Overall, our results show that while the formation of cell-type–specific layers in mouse vLGNe does not depend on RGC inputs, retinal signals are critical for their maintenance.

## Significance Statement

This study investigates the pivotal role of RGC axons in the distribution of GABAergic cells of the mouse ventrolateral geniculate nucleus (vLGN). While the impact of retinal inputs on the classical image-forming vision pathway to the dorsal lateral geniculate nucleus (dLGN) is well-documented, the significance of such inputs in the nonimage-forming vision pathway (vLGN) remains elusive. The research establishes that, contrary to the initial formation of GABAergic laminae, the maintenance of these layers crucially depends on retinal signals. This study advances our understanding of sensory pathway development, unraveling key mechanisms governing neuronal organization in nonimage-forming vision.

## Introduction

In the vertebrate visual system, axons of retinal ganglion cells (RGCs) convey visual information from the world to numerous distinct regions of the hypothalamus, thalamus, and midbrain (Muscat et al., 2003; Morin and Studholme, 2014; Monavarfeshani et al., 2017). In rodents, RGC axons innervate a region of the visual thalamus known as the lateral geniculate complex, which is composed of three nuclei: the dorsal lateral geniculate nucleus (dLGN), the ventrolateral geniculate nucleus (vLGN), and the intergeniculate leaflet (Fleming et al., 2006; Gaillard et al., 2013; Morin and Studholme, 2014; Martersteck et al., 2017; Monavarfeshani et al., 2017). In recent years, anatomical and functional studies have unmasked the complexity of the role of visual thalamus in the processing of visual information, although the bulk of these efforts have focused on dLGN, which connects the retina to the visual cortex and is associated with image-forming vision (Huberman et al., 2008; Guido, 2018; Tiriac et al., 2018).

In dLGN, numerous studies have revealed details about the developmental timeline of thalamocortical relay cells (TRCs), the migration of local interneurons, the arrival of other types of axons, and the emergence and impact of experience-dependent visual activity on each of these processes. During perinatal mouse development, RGC axons establish connections with the dLGN, with axons from the contralateral eye reaching the dLGN between embryonic day 16 and birth (E16–P0), preceding the arrival of axons from the ipsilateral eye (P0–P2; Godement et al., 1980). By the time eye-opening occurs, approximately postnatal day 12 to 14 (∼P12–P14), the eye-specific domains become well-segregated and resemble the mature pattern of innervation seen in adults (Jaubert-Miazza et al., 2005; Huberman et al., 2008). The refinement of these eye-specific domains and the formation of retinotopic maps involves the remodeling of individual axon arbors, where axons projecting to inappropriate target areas are retracted or eliminated, while those projecting to the correct locations are matured and maintained (Dhande et al., 2011). The mechanisms underlying retinotopic map formation and eye-specific axon segregation have been well studied and include complex interactions between developmentally relevant molecular cues and both spontaneous and experience-dependent neural activity (Pfeiffenberger et al., 2005, 2006; Cang et al., 2008; Huberman et al., 2008).

For many years, it has been appreciated that retinal inputs not only convey neural signals from the retina to the dLGN but also influence thalamic development. Once RGC axons arrive in the visual thalamus, they contribute critical roles in shaping the development of several distinct cell types and nonretinal circuits in dLGN. For example, retinal inputs play a crucial role in the morphological development of principal neurons within dLGN, and their loss disrupts the dendritic growth of these neurons (Chen and Regehr, 2000; El-Danaf et al., 2015; Cheadle et al., 2018). Perhaps more surprisingly, retinal inputs are also critical for the recruitment of local GABAergic interneurons in dLGN (Golding et al., 2014; Jager et al., 2016; Charalambakis et al., 2019, Somaiya et al., 2022). Moreover, retinal inputs influence the timing of the arrival of other types of nonretinal axons, including corticogeniculate axons, which are the most abundant axons that innervate thalamocortical relay cells in dLGN (Brooks et al., 2013; Seabrook et al., 2013).

In stark contrast to this, very little is known about the development of cell types and associated circuitry in vLGN. This knowledge gap has been due, in part, to the paucity of what is known about vLGN cytoarchitecture and neuronal cell types. Our recent study accounted for at least six distinct types of GABAergic neurons in vLGN, many of which organize into hidden subtype-specific laminae and appear to receive direct retinal inputs (Sabbagh et al., 2021). Here, we sought to document the development of these vLGN laminae. Based on the critical role of retinal inputs in orchestrating aspects of dLGN development, we asked whether retinal inputs are required for establishing laminae within the retinorecipient vLGN. Surprisingly, we discovered that retinal inputs are not required for the initial formation of vLGN laminae but instead are required for their maintenance.

## Materials and Methods

### Animals

Wild-type C57BL/6 mice were obtained from the Jackson Laboratory. *Math5*^−/−^ mice (stock #042298-UCD; Wang et al., 2001) were obtained from Steven W. Wang (University of Texas MD Anderson Cancer Center). *Calb2^Cre^* and *Shh^fl/fl^* were obtained from the Jackson Laboratory. *Fgf15^−/−^* mice (stock #032840-UCD; Wright et al., 2004) were obtained from S. Kliewer (University of Texas Southwestern Medical Center). Animals were housed in a temperature-controlled environment, in a 12 h dark/light cycle, and with access to food and water *ad libitum*. Both male and female mice were used in the experiments. Genomic DNA was isolated from tails genotyping as previously described (Su et al., 2010) using the following primers: Cre, fwd-CGTACTGACGGTGGGAGAAT, rev-TGCATGATCTCCGGTATTGA; *Shh^fl/fl^*, fwd-CAGAGAGCATTGTGGAATGG, rev-CAGACCCTTCTGCTCATGG; and *Math5^−/−^*, fwd-ATGGCGCTCAGCTACATCAT, rev-GGGTCTACCTGGAGCCTAGC. All animal procedures were performed in accordance with the Virginia Tech Animal Care Committee's regulations. Unless otherwise stated, *n* = number of animals, and a minimum of three age- and genotype-matched animals were compared in all experiments.

### Riboprobe production

Riboprobes against *Lypd1* (Lypd1-F, AAGGGAGTCTTTTTGTTCCCTC; Lypd1-R, TACAACGTGTCCTCTCAGCAGT; NM_145100.4, nucleotides 849−1,522; NM_007492.4, nucleotides 1,830−2,729), *Ecel1* (Ecel1-F, CGCGCTCTTCTCGCTTAC; Ecel1-R, GGAGGAGCCACGAGGATT; NM_001277925.1, nucleotides 942−1,895), *Penk* (Penk-F, TTCCTGAGGCTTTGCACC; Penk-R, TCACTGCTGGAAAAGGGC; NM_001002927.3, nucleotides 312−1,111), and *Arx* (Arx-F, CTGAGGCTCAAGGCTAAGGAG; Arx-R, GGTTTCCGAAGCCTCTACAGTT; NM_007492.4, nucleotides 1,830–2,729) were generated in-house. cDNAs were generated using SuperScript II Reverse Transcriptase First Strand cDNA Synthesis kit (catalog #18064014, Invitrogen) according to the manufacturer’s manual, amplified by PCR using primers designed for the above gene fragments, gel purified, and then cloned into a pGEM-T Easy Vector using pGEM-T Easy Vector kit (catalog #A1360, Promega) according to the kit manual. Antisense riboprobes against target genes were synthesized from 5 μg linearized plasmids using digoxigenin (DIG) or fluorescein-labeled uridylyltransferase (UTP; catalog #11685619910, catalog #11277073910, Roche) and the MAXIscript In Vitro Transcription Kit (catalog #AM1312, Ambion), according to the manufacturer's instructions. Five micrograms of riboprobe (20 μl) were hydrolyzed into ∼0.5 kb fragments by adding 80 μl of water, 4 μl of NaHCO3 (1 M), and 6 μl Na2CO3 (1 M) and incubating the mixture at 60°C. The RNA fragments were precipitated in ethanol and resuspended in RNase-free water.

### ISH

In situ hybridization (ISH) was performed on 16-μm-thin cryosections (Monavarfeshani et al., 2018). Sections were first allowed to air-dry for 1 h at room temperature and washed with PBS for 5 min to remove freezing media. They were then fixed in 4% PFA for 10 min, washed with PBS for 15 min, incubated in proteinase K solution for 10 min, washed with PBS for 5 min, incubated in 4% PFA for 5 min, washed with PBS for 15 min, incubated in acetylation solution for 10 min, washed with PBS for 10 min, incubated in PBS-diluted 0.1% triton for 30 min, washed with PBS for 40 min, incubated in 0.3% H_2_O_2_ for 30 min, washed with PBS for 10 min, prehybridized with hybridization solution for 1 h, and then hybridized with heat-denatured riboprobes at 62.5°C overnight. The sections were then washed five times in 0.2× SSC buffer at 65°C. Slides were then washed with TBS, blocked, and incubated with horseradish peroxidase-conjugated anti-DIG (catalog #11426346910, Roche) or antifluorescent antibodies (catalog #11207733910, Roche) overnight at 4°C. Finally, the bound riboprobes were detected using a tyramide signal amplification system (catalog #NEL753001KT, PerkinElmer).

### Intraocular injection of CTB

We anesthetized mice with isoflurane or hypothermia for intraocular injections. A fine glass pipette attached to a Picospritzer was used to intravitreally inject 1–2 μl of Cholera toxin subunit B (CTB) (1 mg/ml) into the eye. After 2–3 d, the mice were perfused, and their PFA-fixed brains were sectioned (100 μm) using a vibratome (HM650v, Thermo Fisher Scientific), stained with DAPI (1:5,000 in water), and mounted using Vectashield (Vector Laboratories, RRID:AB_2336789).

## Quantification and imaging

### Imaging

All imaging for quantification was performed on a confocal Zeiss LSM 700 microscope at 20× magnification and 0.5 digital zoom. Each representative image in the figure is a maximum-intensity projection. Colocalization was confirmed using single-plane images. Cell counts were obtained from the three sections with the largest overall area within the middle of vLGN, using the hippocampus pyramidal cell layer as a guide for consistency between animals. When comparing sections from different age groups or genotypes, images were acquired with identical parameters. A minimum of three animals (per genotype and per age) were compared in all experiments.

### Area of vLGNe and vLGNi

**Figure 1.**
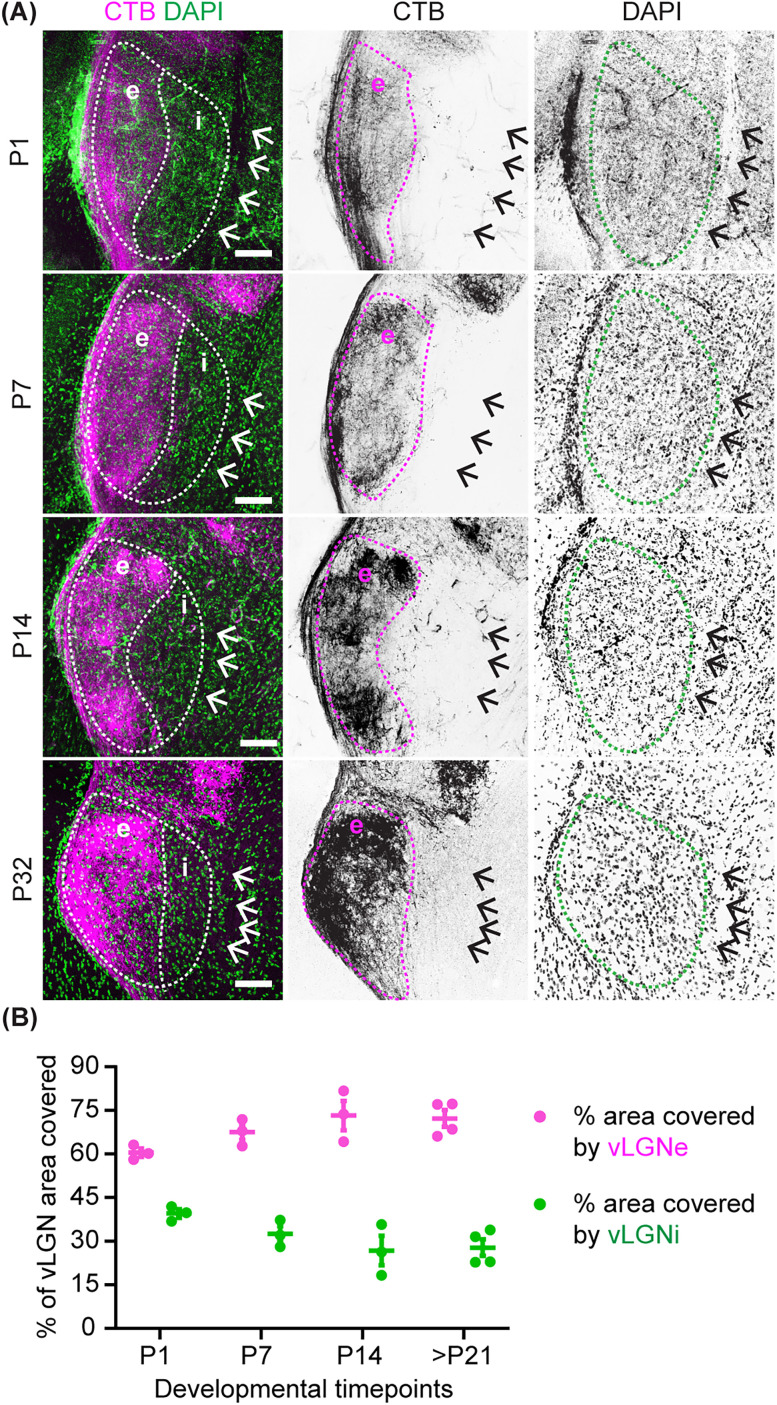
Developmental expansion of vLGNe coincides with the arrival and maturation of retinal inputs. ***A***, Retinal terminals in vLGNe labeled by monocular injection of fluorescently conjugated CTB at P1, P7, P14, and P32 of the wild-type (WT) thalamus. The medial border of vLGNi was determined by cytoarchitecture (see black arrows in rightmost panels). ***B***, Quantification of the percentage of vLGN area covered by vLGNe and vLGNi across developmental time points in WT mice. Each data point represents one biological replicate and bars depict means ± SEM (P1, P7, P14 *n* = 3; >P21 *n* = 4). Scale bar: ***A***, 100 μm.

To measure the area of vLGNe and vLGNi in wild-type and *Math5*^−/−^ animals, the entire vLGN was traced with ImageJ software (version 1.52n, NIH), and the total area was measured. For this purpose, the lateral boundary of the vLGN and vLGNe was defined by the optic tract, and the change in density of DAPI^+^ cells was used to discern the medial border of vLGNi (Fig. 1*A*, arrows) since previously identified markers that exclusively label neurons in vLGNi do not reliably label cells in the neonatal brain (data not shown). The medial border of vLGNe was identified by discerning the boundary of CTB localization. The area of vLGNi was determined by subtracting the previously determined area of vLGNe from the overall area of vLGN.

### Quantification of laminar organization

To quantify the spatial distribution of cell-type marker expression across the entire vLGN, we used our previously published line scan script, khatscan, that runs in ImageJ and overlays the vLGN with equally spaced lines (Sabbagh et al., 2021). A summary of how this script works: confocal images are first thresholded and binarized to determine the curvature of the vLGN in a particular image, the script prompts the user to draw a line along the optic tract adjacent to the vLGN, then lines of a set length and number are automatically drawn guided by that curve and plots detected signal across the *x*-coordinates of each line. Signal values at each *x*-coordinate are then averaged to determine where there is specific enrichment for that marker in that spatial zone of the vLGN.

### Percentage of *Ecel1^+^*, *Lypd1^+^*, and *Penk^+^* cells

Images were obtained using 20× magnification and 0.5 digital zoom. We quantified *Ecel1*^+^, *Lypd1*^+^, and *Penk*^+^ (by ISH) cells and divided the total number of DAPI^+^ cells counted in that tissue section. The “Count Tool” function in Adobe Photoshop (version 21.1.2) was utilized for counting purposes. Summary graphs include individual data along with mean ± SEM values. Statistical analyses (Student's *t* test) were performed using GraphPad Prism (version 8.0). *p* < 0.05 values were considered to be significantly different.

## Results

### Development of the external and internal divisions of vLGN

The rodent vLGN can be divided into two distinct domains based on cytoarchitecture: the retinorecipient external division (vLGNe) and the nonretinorecipient internal division (vLGNi). Our recent study identified and characterized novel GABAergic neuronal types that occupy these regions (Sabbagh et al., 2021). To investigate the development of the cell types in the neo- and perinatal vLGN, we assessed cytoarchitectural changes in these vLGN subdivisions during the first 3 weeks of mouse development. We identified these regions based on DAPI labeling and anterograde labeling of retinal axons. Retinal terminals were labeled by intraocular injection of fluorescently conjugated CTB, a tracer that efficiently labels retinal projections into the brain (Muscat et al., 2003). The area occupied by retinal axons was determined to be vLGNe (Guido, 2018; Sabbagh et al., 2021; Govindaiah et al., 2023). We observed that the proportion of vLGN represented by vLGNe increased with postnatal age, while the percentage of vLGN area represented by vLGNi decreased (Fig. 1*A,B*). At P1, the proportions of the area occupied by vLGNe and vLGNi were 60 and 40%, respectively. After P21, the difference between the proportions of vLGNe and vLGNi doubled, with vLGNe occupying 72% of vLGN and vLGNi occupying only 28% of vLGN. This suggests that the arrival of retinal inputs potentially influences the expansion of vLGNe during development.

### Subtype-specific laminae are specified in the neonatal vLGN

**Figure 2.**
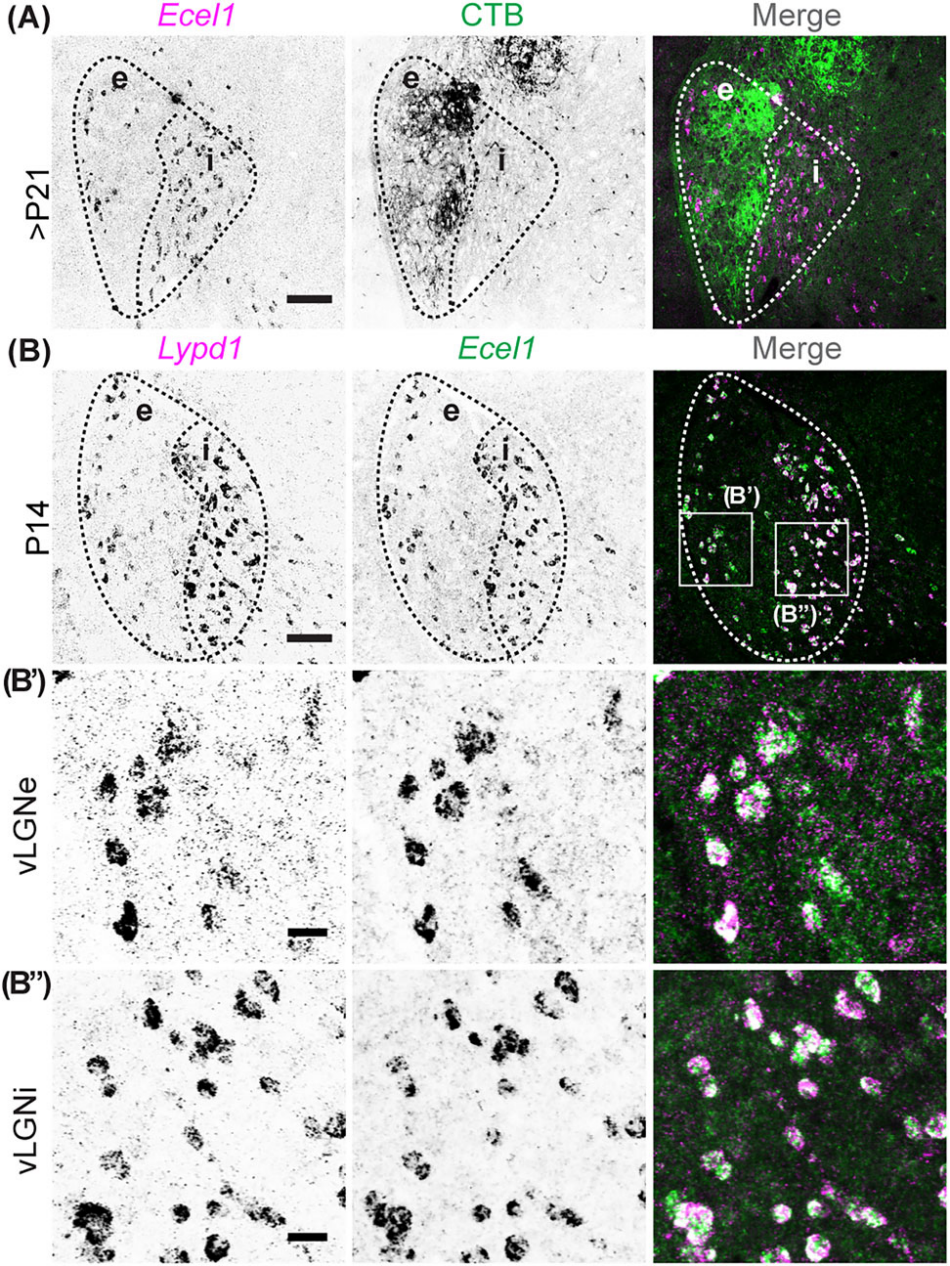
Molecular markers to identify the boundaries of vLGNe and vLGNi. ***A***, ISH with riboprobes against *Ecel1* following intraocular injection of fluorescently conjugated CTB in >P21 WT vLGN. ***B***, Double ISH with riboprobes against *Lypd1* and *Ecel1* in P14 WT vLGN. ***B’*** depicts vLGNe. ***B”*** depicts vLGNi. Scale bars: ***A***, ***B***, 100 μm; ***B’***, ***B”***, 20 μm.

We next set out to characterize the development of neuronal cell types in vLGN and explore when cell-type–specific laminae form in vLGN. Our previous study characterized several vLGN cell types based on the generation of specific transcripts. vLGNe can be further divided into at least four subtype-specific layers, each composed of molecularly and functionally distinct neurons, several of which receive direct monosynaptic input from the retina (Sabbagh et al., 2021). In some cases, the expression of these transcripts was shown to be developmentally regulated, making it potentially challenging to use riboprobes against those genes to study laminar organization at different developmental ages. This was the case for both *Ecel1^+^* and *Lypd1^+^* GABAergic neurons, which are present in the same region (Fig. 2*A,B*) within the vLGN but exhibited different developmental expression patterns, with *Lypd1* expression decreasing with development and *Ecel1* expression being low or not detectable at early postnatal ages (Sabbagh et al., 2021). To determine if these markers were generated in the same subtype of GABAergic cells and only exhibited different developmental expression patterns, we performed double ISH at P14, a developmental period when both genes are expressed high in vLGN. Indeed, our results showed that all cells that express *Lypd1* mRNA also generate *Ecel1* mRNA in vLGNe and vLGNi (Fig. 2*B–B”*), indicating that these markers correspond to the same GABAergic neuronal subtype.

**Figure 3.**
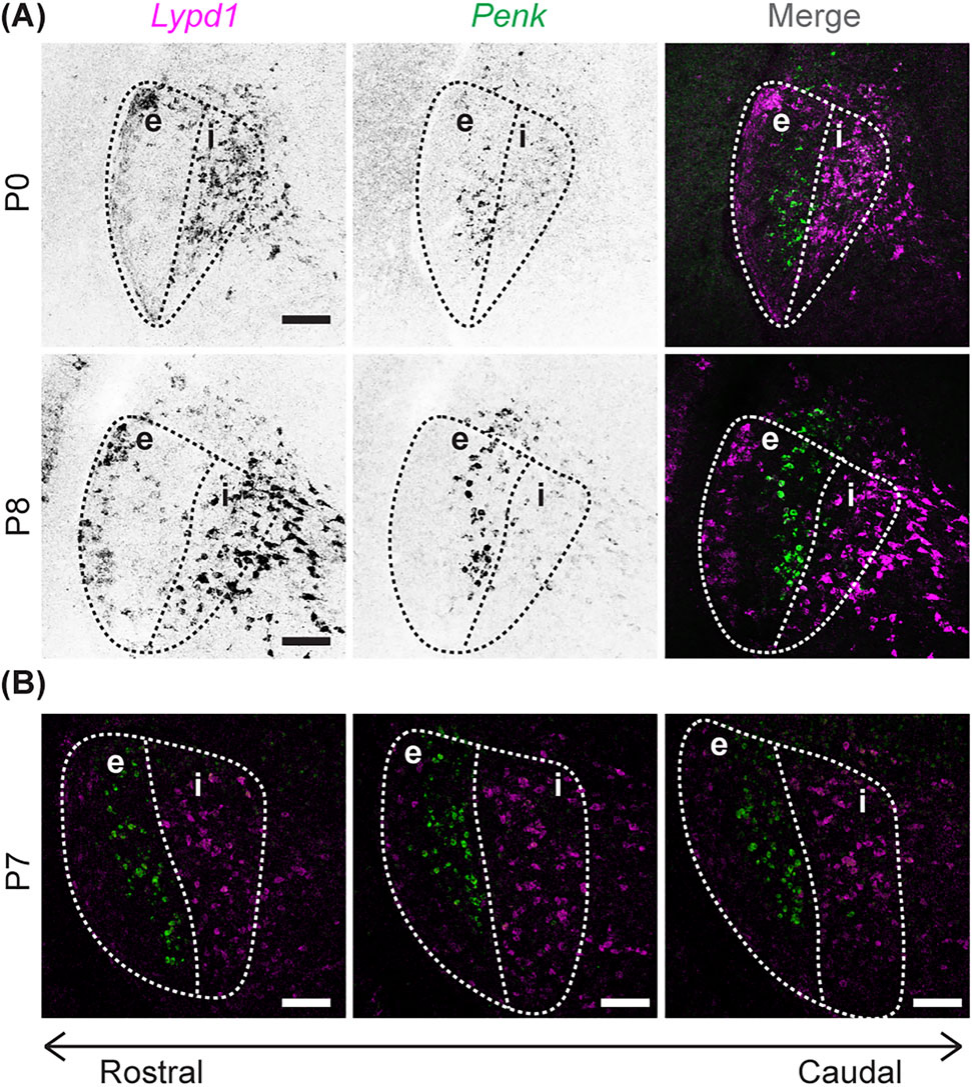
Subtype-specific sublaminae are specified in the neonatal vLGN. ***A***, Double ISH with riboprobes against *Lypd1* and *Penk* at P0 and P8 in WT vLGN. ***B***, Double ISH with riboprobes against *Lypd1* and *Penk* at P7 in WT vLGN along the rostocaudal axis. Scale bar, 100 μm.

To begin to elucidate whether the transcriptionally distinct laminae in vLGN were expressed during early development, we studied the development of *Ecel1^+^*/*Lypd1^+^* and *Penk1^+^* neurons. In adult mice, *Ecel1*^+^ cells occupy the lateral-most border of vLGNe and the entire vLGNi, while *Penk*^+^ cells occupy the medial-most border of vLGNe (Sabbagh et al., 2021). Interestingly, our double ISH results showed that the distinct spatial localization of *Lypd1*^+^ cells and *Penk*^+^ cells was present as early as P0 in the vLGN (Fig. 3*A*) and was present throughout the longitudinal axis (Fig. 3*B*). These data suggest that the formation of cell-type–specific laminae in vLGN occurs during embryonic development.

### Loss of retinal inputs disrupts vLGN cytoarchitecture lamination

**Figure 4.**
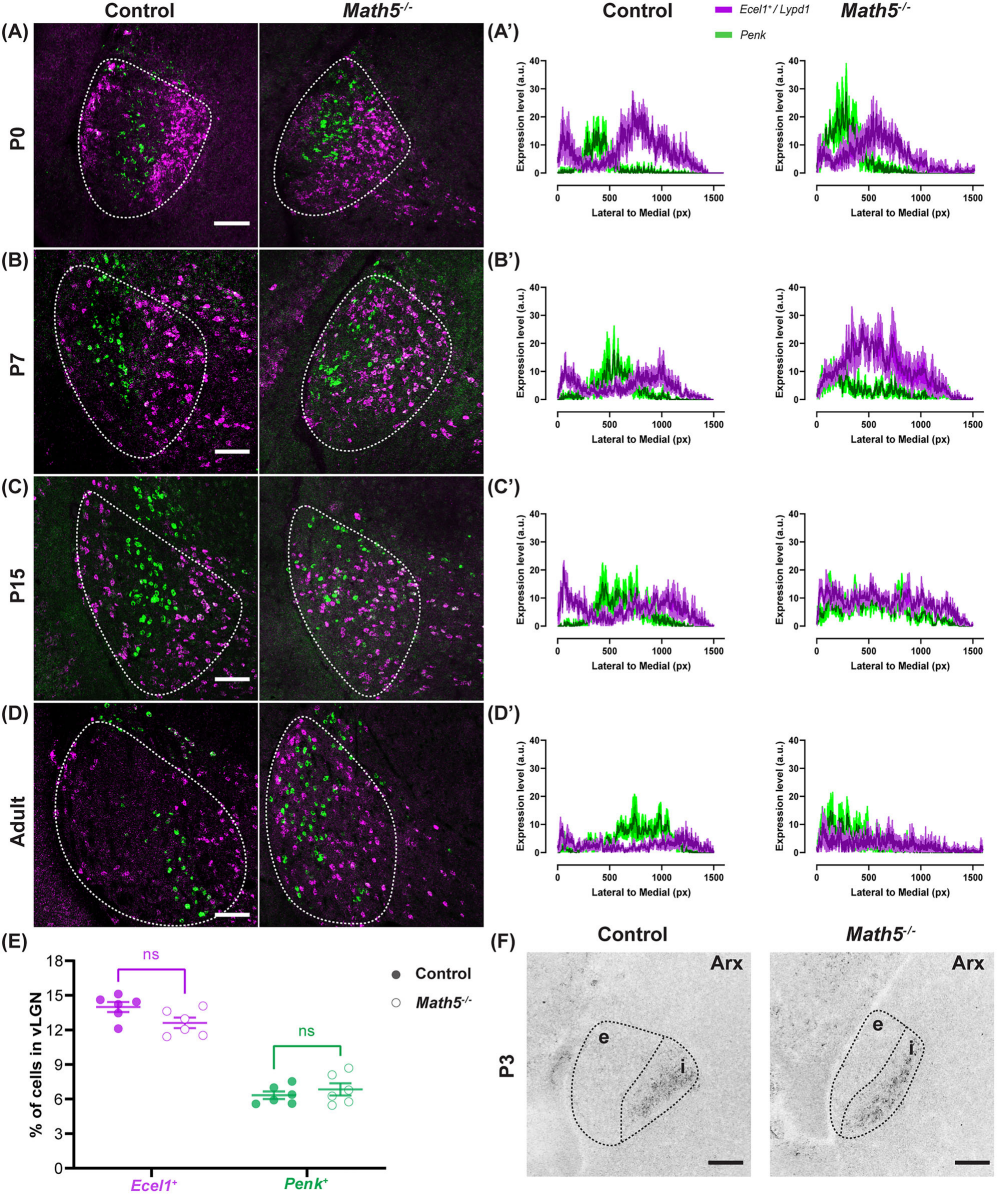
Loss of retinal inputs leads to progressive disruption of vLGN lamination. ***A***, Double ISH with riboprobes against *Lypd1* and *Penk* in P0 WT and *Math5*^−/−^ mice. ***B–D***, Double ISH with riboprobes against *Ecel1* and *Penk* in (***B***) P7, (***C***) P15, and (***D***) adult WT and *Math5*^−/−^ mice. ***A’***, Line scan analysis of the spatial distribution of *Lypd1*^+^ and *Penk*^+^ cells. Arbitrary fluorescence units (a.u.) are plotted against the distance from the lateral-most part of the vLGN to the most medial by pixel (px). The solid line represents the mean, and the shaded area represents SEM (*n* = 3). ***B’–D’***, Line scan analysis of the spatial distribution of *Ecel1*^+^ and *Penk*^+^ cells. Arbitrary fluorescence units (a.u.) are plotted against the distance from the lateral-most part of the vLGN to the most medial by pixel (px). The solid line represents the mean, and the shaded area represents SEM (*n* = 3). ***E***, Quantification of the percentage of *Ecel1*- and *Penk*-expressing cells in vLGN of P14 WT and *Math5*^−/−^ mice. Each data point represents one biological replicate and bars depict means ± SEM. ns indicates no significant differences by Student’s *t* test (*n* = 6). ***F***, ISH with riboprobes against *Arx* in P3 WT and *Math5*^−/−^ mice. Scale bar: ***A–D***, ***F***, 100 μm.

The existence of distinct GABAergic laminae in the retinorecipient vLGNe, but not in vLGNi, suggests that retinal inputs might be involved in the lamination of vLGN. Previous studies have found that retinal inputs direct spacing and migration of GABAergic interneurons in the dLGN (Golding et al., 2014; Charalambakis et al., 2019; Somaiya et al., 2022). Therefore, we asked whether retinal inputs are critical for specifying the laminar organization of GABAergic neurons in vLGN by utilizing *Math5^−/−^* mice that lack RGCs and their central projections in the brain (Wang et al., 2001; Brooks et al., 2013; Seabrook et al., 2013; Hammer et al., 2014). To test this, we used double ISH with riboprobes against *Penk* at all ages and either *Lypd1* at ages up to P5 or *Ecel1* for ages >P5. We observed that GABAergic laminae exist in *Math5*^−/−^ vLGN at birth and the spatial localization of *Ecel1^+^/Lypd1^+^ and Penk^+^* cells is very similar to WT controls (Fig. 4*A*). We also performed ISH with riboprobe against *Arx*, a marker for vLGNi (Sabbagh et al., 2021), and found that the division between retinorecipient vLGNe and nonretinorecipient vLGNi in *Math5*^−/−^ vLGN is similar to WT controls (Fig. 4*F*). Interestingly, the separation in the spatial distribution of *Ecel1^+^*/*Lypd1^+^* and *Penk^+^* neurons within vLGN decreased dramatically by eye-opening (Fig. 4*B,C*). To quantitatively assess the differences in the laminar organization of GABAergic neurons between control and *Math5*^−/−^ mice, we performed line scan analysis in ImageJ to measure binarized signal corresponding to *Ecel1^+^*/*Lypd1^+^* and *Penk^+^* neurons along the mediolateral axis of vLGN. Indeed, our data showed that while *Ecel1^+^*/*Lypd1^+^* and *Penk^+^* laminae exist at birth in *Math5*^−/−^ mice (Fig. 4*A’*), these GABAergic subtypes begin to lose their spatial localization after the first week of postnatal development (Fig. 4*B’,C’*) and by eye-opening are occupying overlapping territory in vLGN (Fig. 4*D’*).

The disrupted organization could be a result of the lack of retinal input-dependent cues (molecules and/or intrinsic activity) that are required to maintain laminar arrangement or cues required for the survival of vLGNe neurons. To explore the latter possibility, we next determined whether there were changes in the percentage of different GABAergic subtypes in the absence of the retinal inputs. We assessed the percentage of cells rather than the cell density due to the significant difference in total vLGN area and cell count between control and *Math5*^−/−^ LGN (Hammer et al., 2014; El-Danaf et al., 2015; Charalambakis et al., 2019). By quantifying *Ecel1^+^*/*Lypd1^+^* and *Penk^+^* neurons, our results show no change in their percentage between control and *Math5*^−/−^ vLGN (Fig. 4*E*). Taken together, these data suggest that retinal inputs are not impacting projection neuron number in vLGN or the initial formation of cell-type–specific laminae in vLGN but instead are essential for the maintenance of cell-type–specific layers in vLGNe.

### Loss of retinal sonic hedgehog and *Fgf15* does not disrupt subtype-specific organization in vLGN

**Figure 5.**
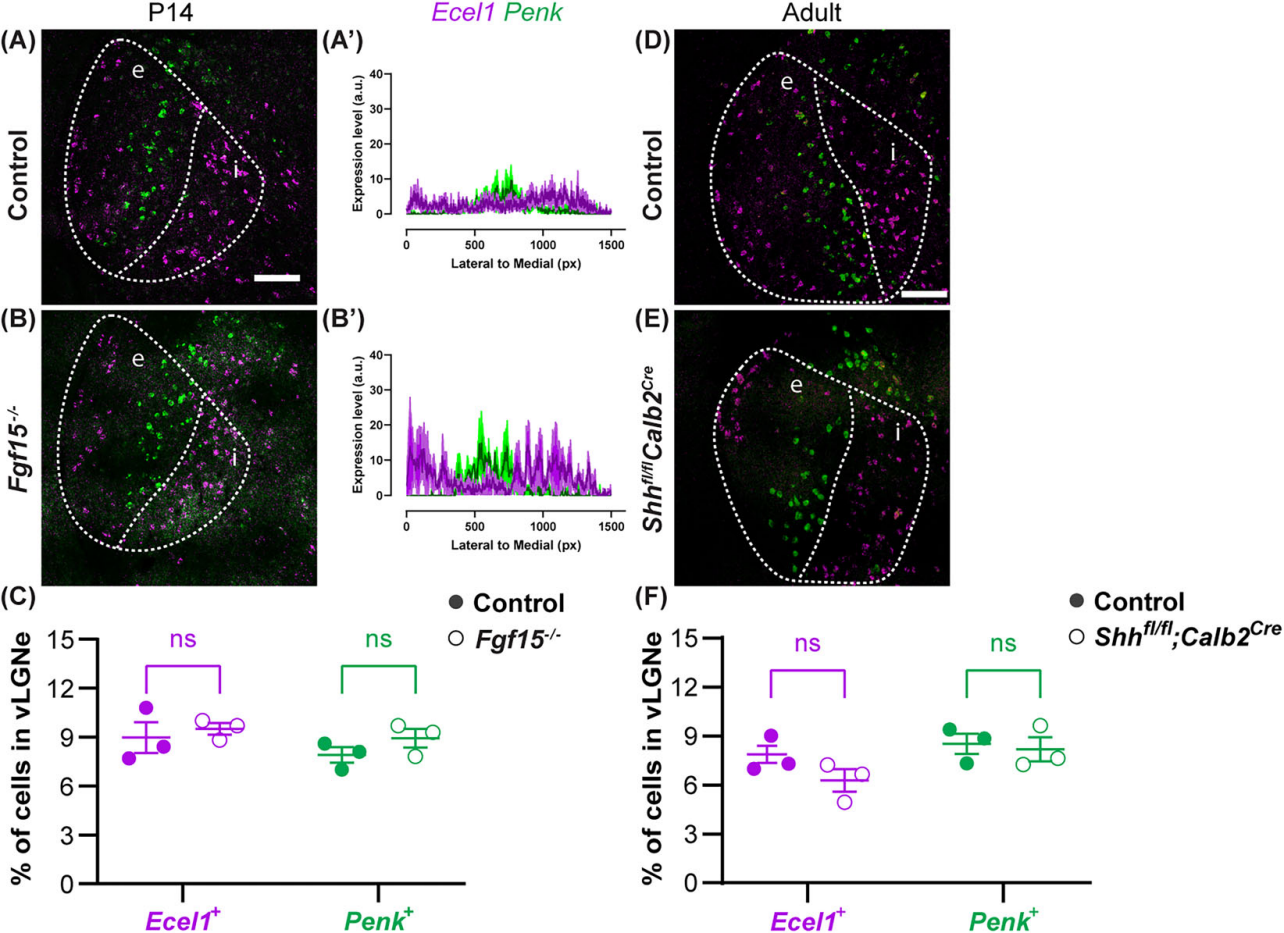
Loss of retinal SHH and *Fgf15* does not disrupt the subtype-specific organization in vLGN. ***A***, ***B***, Double ISH with riboprobes against *Ecel1* and *Penk* in P14 WT and *Fgf15*^−/−^ mice. ***A’***, ***B’***, Line scan analysis of the spatial distribution of *Ecel1*^+^ and *Penk*^+^ cells. Arbitrary fluorescence units (a.u.) are plotted against the distance from the lateral-most part of the vLGN to the most medial by pixel (px). The solid line represents the mean, and the shaded area represents SEM (*n* = 3). ***C*,** Quantification of the percentage of *Ecel1*- and *Penk*-expressing cells in vLGN of P14 WT and *Fgf15*^−/−^ mice. Each data point represents one biological replicate and bars depict means ± SEM. ns indicates no significant differences by Student’s *t* test (*n* = 3). ***D***, ***E***, Double ISH with riboprobes against *Ecel1* and *Penk* in adult WT and *Shh^fl/fl^Calb2^Cre^* mice. ***F***, Quantification of the percentage of *Ecel1*- and *Penk*-expressing cells in vLGN of adult WT and *Shh^fl/fl^Calb2^Cre^* mice. Each data point represents one biological replicate and bars depict means ± SEM. ns indicates no significant differences by Student’s *t* test (*n* = 3). Scale bar: ***A***, ***B***, 100 μm.

Recent studies revealed that RGCs release sonic hedgehog (SHH) to induce the expression of fibroblast growth factor 15 (FGF15), which is required for the recruitment of GABAergic interneurons into dLGN and vLGN (Su et al., 2020, Somaiya et al., 2022). Given that the absence of retinal inputs disrupts vLGN cytoarchitecture lamination, we wondered whether SHH and FGF15 are required for the maintenance of cell-type–specific layers in vLGNe. To test this, we performed double ISH with riboprobes generated against *Ecel1* and *Penk* mRNA in control, *Fgf15*^−/−^, and *Shh^fl/fl^Calb2^Cre^* mice, where RGCs lack SHH expression (Somaiya et al., 2022). We observed that neurons generating *Ecel1* and *Penk* mRNA retained their laminar identities in both the *Fgf15*^−/−^ mice (Fig. 5*A,B*) and the *Shh^fl/fl^Calb2^Cre^* mice (Fig. 5*D,E*), which was confirmed with the quantitative assessment via line scans (Fig. 5*A’,B’*). We also found no change in the percentage of *Ecel1*^+^ and *Penk*^+^ cells between control and *Fgf15*^−/−^ vLGN (Fig. 5*C*) or *Shh^fl/fl^Calb2^Cre^* vLGN (Fig. 5*F*). These findings indicate that the developmental influence of FGF15 via the SHH pathway is not critical for the recruitment and distribution of *Ecel1*^+^ and *Penk*^+^ cells in vLGN, nor is it necessary for the maintenance of these GABAergic laminae.

## Discussion

In this study, we characterized the molecular markers labeling distinct subtypes of GABAergic neurons in vLGN during early postnatal development. Using *Ecel1*^+^ and *Penk*^+^ neurons, we found that cell-type–specific lamination in vLGNe is determined during embryonic and/or neonatal development, before the migration of local interneurons, the arrival of other axons, eye-opening, and the emergence of experience-dependent visual activity. In mutant mice with no RGC axons, we found that retinal inputs are required for the maintenance of vLGN cytoarchitecture organization but are not required for the formation of these laminae. Lastly, we demonstrated that this redistribution of principal GABAergic cells is not due to the loss of FGF15, an RGC-induced, astrocyte-derived guidance cue essential for the migration of interneurons into rodent LGN (Su et al., 2020, Somaiya et al., 2022). Together, these data provide insights into the role of sensory inputs in the development of vLGN cytoarchitecture.

Previous studies have shown that retinal inputs are necessary for the migration of GABAergic interneurons into the visual thalamus (Seabrook et al., 2013; Somaiya et al., 2022). However, our results show that retinal inputs are not required for the migration of vLGN GABAergic cell types explored in this study, suggesting to us that *Ecel1*^+^ and *Penk*^+^ cells in vLGN are not interneurons and instead may represent projection neurons. In contrast, our previous studies have demonstrated that GFP-labeled interneurons in the GAD67-GFP mouse line do require retinal inputs to properly migrate into vLGN (Su et al., 2020). Taken together, these data begin to suggest that retinal inputs exert different influences on principal (projection) neurons than on local interneurons in vLGN. This is certainly the case in dLGN, although projection neurons in dLGN are excitatory and not GABAergic.

The presence of *Lypd1*^+^ and *Penk*^+^ cells in vLGN, both labeling distinct subpopulations of neurons as early as P0, indicated to us that these GABAergic neurons have already migrated into lamina of the vLGN as retinal axons first innervate this region. While *Lypd1*^+^ and *Penk*^+^ neurons did in fact stratify into these laminae, the layers they formed appeared to be less refined in the early developing vLGN (Fig. 4*A–D*). For instance, in the adult vLGN, *Penk*^+^ neurons formed a more sharpened sublamina that is more clearly delineated (Fig. 4*D*), whereas at P0, *Penk*^+^ neurons are still layer-forming but appear to be more widespread and occupy more of the lateromedial space of the vLGN than during adulthood (Fig. 4*A*). These differences suggest that this laminar organization continues to refine after birth. Since our results indicate that retinal inputs are not required for their formation but are necessary, retinal input-derived cues might be important for the refinement of GABAergic laminar organization in vLGN.

While previous studies have determined that astrocyte-derived FGF15 is essential for the recruitment of GABAergic interneurons, the loss of functional FGF15 did not reproduce the defects in subtype-specific lamination associated with the loss of retinal input. Thus, proper maintenance of vLGN lamination in the presence of retinal inputs could be due to other developmentally regulated factors released by RGC axons or retinal activity. Indeed, RGCs in the retina undergo significant transcriptomic changes during development, suggesting that molecules expressed after eye-opening could be potentially important for vLGN lamination (Shekhar et al., 2022). Another possibility is that retinal input is necessary for the proper development of vLGN ECM rather than principal neuron lamination. To assess if disruption of retinal inputs impairs ECM formation and maintenance, which subsequently impairs the location of principal neurons in vLGN, it would be beneficial to evaluate chondroitin sulfate proteoglycans that are known to be enriched in vLGN, such as phosphacan (Brooks et al., 2013). The rearrangement of GABAergic cell laminae might also be an indirect consequence of the absence of retinal inputs regulating collicular and/or cortical input to the vLGN. Although such functions of retinal inputs have been recognized in the dLGN, they remain unexplored in the context of the vLGN. Investigating the impact of cortical and collicular inputs by conducting experiments where these neurons are silenced could yield valuable insights.

Overall, our study suggests two distinct developmental processes involved in specifying vLGN laminae: one to form a layer and the other to refine and sharpen it. Thus, the cells of vLGNe and vLGNi are genetically hardwired to be retinorecipient or nonretinorecipient, respectively, as opposed to assuming cellular phenotypes because of retinal input. Our data, when taken together, suggest the possibility that the functional organization of nonimage-forming information from the retina to vLGN is extracted from the segregation of transcriptionally distinct retinorecipient cells. We view these results as a framework for further dissecting the structure, circuitry, and functions of the vLGN at a cell-type–specific level. How the heterogeneity and organization contribute to the yet-to-be-determined functions of the vLGN remains to be defined.

## Synthesis

Reviewing Editor: Lynda Erskine, University of Aberdeen Decisions are customarily a result of the Reviewing Editor and the peer reviewers coming together and discussing their recommendations until a consensus is reached. When revisions are invited, a fact-based synthesis statement explaining their decision and outlining what is needed to prepare a revision will be listed below. The following reviewer(s) agreed to reveal their identity: Alessio Delogu, Samer Hattar.

Thank you for submitting your interesting work to eNeuro. As you will see from the review synthesis below, there is enthusiasm from the reviewers for publishing work in eNeuro subject to appropriate revisions being made. These revisions should be able to be addressed through text and figure changes. The reviewer also have some OPTIONAL suggestions for additional experiments that would strengthen the conclusions of the paper (or form the basis for a future study).

General comments from the reviewers:

In this article the authors use the Math5 loss of function mutant to assess the developmental role of retinal innervation in the establishment and maintenance of neuronal laminae in the LGv. Previously characterised molecular markers are used to map at the histological level the distribution of defined GABAergic populations.

The findings support the view that within the lateral geniculate complex, GABA projection neurons differ substantially from local circuit GABA interneurons. While the fine distribution of GABA interneurons depends on retinal innervation (via Shh-induced astrocyte expression of Fgf15), the laminar distribution of projection GABA neurons does not. However, a late requirement for the retinal afferentiation in maintenance of the laminar organisation is described. This is perhaps not too surprising, given that interneurons must undergo a complex sequence of radial/tangential/radial migration to populate the LGN, while projection GABA neurons likely require only radial migration from the respective progenitor domain in prosomere 3 (prethalamus).

This manuscript investigates the timeline of vLGN laminae development and their dependence on retina input. The manuscript shows that vLGN laminae develop independent of retinal input but become disorganized overtime without retinal input to maintain their structure. In addition, the authors show that vLGN laminae maintenance does not require Fgf15.

Requested revisions:

1. In order to strengthen the conclusions of the paper the authors should provide more images along the rostro-caudal length of the LGv with relative quantifications for all figures. This is because the histological architecture changes significantly along the longitudinal dimension.

2. Inclusion of a discussion that includes the possibility of an indirect role of the retinal input in maintenance of LGv lamination that is mediated by descending collicular and/or cortical input to the LGv. Both these sources of LGv innervation would likely be affected in the Math5 mutant and could explain the delayed effect on LGv organisation.

3. In the abstract the authors write that "retinal inputs play important roles in in the development of the dLGN, including... the recruitment of inhibitory interneurons from progenitor zones". Given the current evidence, it is unlikely that progenitor domains active in the ventricular zone at ~E11.5 in the mouse would be under the control of retinal afferentiation. The retinal role is more likely to control the final step of the migration related to the entry and the distribution of the interneurons within the lateral geniculate nuclei.

4. On page 7 line 13 and following from there. The authors correctly refer to the reduced volume of the visual thalamus in the Math5 mutants, but few lines later say: "taken together these data suggest that retinal inputs are not impacting GABAergic cell proliferation, recruitment, or survival in vLGN". If so, the reduced volume of the LGN (including LGv which is virtually all GABAergic) would be difficult to explain. The authors should re-assess their statement.

5. This paper builds on previous work from Sabbagh et al. 2021 by investigating the development and RGC-dependence of Ecel1+ and Penk+ laminae. It would be good to see additional cell markers that make clear laminae that are not included here (specifically Pvalb and/or Spp1).

6. In Figure 4: It looks like the markers Ecel1 and Penk overlap in the mutant mice and not in the control mice (especially mutant panels B and C). The quantification in E does not show/account for this. Please comment and/or quantify this.

Additional points that could be addressed in a revision:

7. Assuming that the authors would have access to the tissue from the RGC-specific deletion of Shh (Somaiya et al., eLife 2022), they could include this material in the manuscript in the same way that they have looked at the Fgf15 loss of function material. While in the LGd, Shh was shown to act by activating Fgf15 expression, there remains the possibility that in the LGv, retinal input acts via Shh to sustain lamination, independent of Fgf15.

8. The manuscript shows that without retinal innervation during development, the vLGN laminae that originally formed lose their organization.

a) Is retinal ganglion cell innervation alone, without visual input, sufficient to maintain laminae?

E.g. silence RGCs with tentanus toxin at P0 and investigate vLGN organization in adult.

b). Is there a critical window for RGC-maintenance of the vLGN laminae?

E.g. Does enucleation during adulthood result in the same disorganization of vLGN lamine?

## Author Response

We thank the reviewers for constructive feedback regarding our manuscript. To address their concerns, we have made several changes to our manuscript. This includes changes to the figures and to the manuscript itself. Below, we highlight reviewers' comments in black and our responses in red.

Requested revisions:

1. In order to strengthen the conclusions of the paper the authors should provide more images along the rostro-caudal length of the LGv with relative quantifications for all figures. This is because the histological architecture changes significantly along the longitudinal dimension.

Yes, the structure and size of the visual thalamus (including both the dLGN and vLGN) changes along the rostro-caudal axis in coronal sections. When selecting representative images for vLGN in this manuscript (similar to Sabbagh et al. 2021), we choose the section with the largest overall area. To help address the reviewer's concerns, we have now included additional images in Figure 3 to demonstrate the presence of cell type-specific laminae in multiple sections along the rostro-caudal axis in coronal sections. We have also referred to previous work that also identified these lamina in horizontal images of vLGN (Sabbagh et al., 2021).

2. A discussion that includes the possibility of an indirect role of the retinal input in maintenance of LGv lamination that is mediated by descending collicular and/or cortical input to the LGv should be included. Both these sources of LGv innervation would likely be affected in the Math5 mutant and could explain the delayed effect on LGv organization.

We also agree with this idea that there may be an indirect effect from collicular and/or cortical input to vLGN impacting the rearrangement of GABAergic cell laminae in the absence of retinal input. This information is now included in the discussion section of the revised manuscript.

3. In the abstract the authors write that "retinal inputs play important roles in the development of the dLGN, including... the recruitment of inhibitory interneurons from progenitor zones". Given the current evidence, it is unlikely that progenitor domains active in the ventricular zone at ~E11.5 in the mouse would be under the control of retinal afferentiation. The retinal role is more likely to control the final step of the migration related to the entry and the distribution of the interneurons within the lateral geniculate nuclei.

Thank you for this suggestion. We have now changed the wording to more accurately describe what is known for interneuron migration into visual thalamus.

4. On page 7 line 13 and following from there. The authors correctly refer to the reduced volume of the visual thalamus in the Math5 mutants, but few lines later say: "taken together these data suggest that retinal inputs are not impacting GABAergic cell proliferation, recruitment, or survival in vLGN". If so, the reduced volume of the LGN (including LGv which is virtually all GABAergic) would be difficult to explain. The authors should reassess their statement.

This is an excellent point and we have now revised this statement (to focus on GABAergic projection neurons).

However, based on previous studies in dLGN, which also shrinks in Math5-/- mutants, it is thought that the reduction in size is due to the loss of retinal inputs, the loss of dendritic growth and expansion, and a reduction in the number of local interneurons. We have updated the text to reflect this point.

5. This paper builds on previous work from Sabbagh et al. 2021 by investigating the development and RGC-dependence of Ecel1+ and Penk+ laminae. It would be good to see additional cell markers that make clear laminae that are not included here (specifically Pvalb and/or Spp1).

We appreciate the reviewer's suggestion regarding the investigation of Pvalb+ and Spp1+ cell lamination in Math5-/- mutants, as it would indeed provide valuable insights. However, our study specifically focused on early developmental lamination of vLGN GABAergic cells and, given that both Pvalb+ and Spp1+ exhibit low expression levels until later postnatal ages (Sabbagh et al., 2021), we chose to concentrate on Ecel1+ and 2 Penk+ neurons. We acknowledge the importance of exploring the influence of retinal inputs on other GABAergic cell types in future studies, which could further enrich our understanding of vLGN development.

However, to address the reviewers' comments we have now examined the neonatal expression of Arx, a transcript whose expression is confined to cells in the vLGNi. Here we now show that at P3, Arx expression is already confined to cells in the vLGNi. We further show that the vLGNi-specific expression of Arx persists in Math5-/- mutants (Fig 4F), further suggesting that retinal inputs are not required for the initial specification of vLGN sublamina or subdivisions.

6. In Figure 4: It looks like the markers Ecel1 and Penk overlap in the mutant mice and not in the control mice (especially mutant panels B and C). The quantification in E does not show/account for this. Please comment and/or quantify this.

Yes, we agree with the reviewer that in this example there are some cells that appear to express both markers.

The same is true in controls (for cells that express lower levels of Penk). In more recent studies, we are trying to analyze and quantify this with single nucleus transcriptomic sequencing. Those studies are still in their infancy and we view them as beyond the scope of the current study.

Additional points:

7. Assuming that the authors would have access to the tissue from the RGC-specific deletion of Shh (Somaiya et al., eLife 2022), they could include this material in the manuscript in the same way that they have looked at the Fgf15 loss of function material. While in the LGd, Shh was shown to act by activating Fgf15 expression, there remains the possibility that in the LGv, retinal input acts via Shh to sustain lamination, independent of Fgf15.

We agree with the reviewers that investigating changes in lamination of Shhfl/fl;Calb2Cre tissue further strengthens our findings. This information is now included in Figure 5 and in the discussion section of the revised manuscript.

8. The manuscript shows that without retinal innervation during development, the vLGN laminae that originally formed lose their organization. a) Is retinal ganglion cell innervation alone, without visual input, sufficient to maintain laminae? E.g. silence RGCs with tetanus toxin at P0 and investigate vLGN organization in adults. b). Is there a critical window for RGC-maintenance of the vLGN laminae? E.g. Does enucleation during adulthood result in the same disorganization of vLGN laminae? These are both excellent suggestions. Investigating if this is visual activity driven or solely retinal input influenced process would be an interesting and important future experiment. We also agree with the reviewer's suggestion of doing enucleation during adulthood leading to interesting results. As present, we view such experiments as beyond the scope of the current study.
